# Evaluation of the effectiveness of the human-baited double net traps and BG traps compared with the human landing catches for collecting outdoor *Aedes albopictus* in China

**DOI:** 10.1186/s12889-023-16940-w

**Published:** 2023-10-11

**Authors:** Yuyan Wu, Juan Hou, Qinmei Liu, Jinna Wang, Tianqi Li, Mingyu Luo, Zhenyu Gong

**Affiliations:** Zhejiang Provincial Center for Disease Control and Prevention, Hangzhou City, Zhejiang Province China

**Keywords:** Dengue Fever, *Aedes albopictus*, Monitoring, Human landing catch (HLC), Human-baited double net (HDN), BG trap

## Abstract

Dengue fever is one of the biggest threats to public health in China, causing huge disease burden and economic loss. *Aedes*-mosquito surveillance could be a cornerstone for predicting the risk of *Aedes*-borne diseases and evaluating the effect of vector management during diseases outbreaks. The human landing catch (HLC) method is regarded as the “gold standard” for catching *Aedes* mosquitoes, but it potentially exposes field professionals to vectors of known or unknown pathogens. Human-baited double net (HDN) was recommended to replace HLC for emergency monitoring in China when *Aedes*-borne diseases break out, but it had been reported with low efficiency for capturing *Aedes* mosquitoes. In this study, we compared HLC with HDN and BG traps for field *Aedes albopictus* monitoring, with the aim of evaluating the effectiveness of HDN replacing HLC and finding an effective and safe alternative to the HLC for monitoring *Aedes albopictus*. Six sites in Hangzhou, Shaoxing, and Yiwu, Zhejiang Province, China, were chosen to conduct outdoor HLC, HDN, and BG trap catches from June to October 2021. The tests were performed 3 h apart: 8:30–9:30 AM, 16:30–17:30 PM, and 17:30–18:30 PM. A total of 2330 adult mosquitoes were collected, and *Aedes albopictus* was the most abundant species in all three catches with 848(98.95%), 559(97.39%) and 867 (96.44%) caught in HLC, HDN and BG traps respectively. Compared to HLC, HDN collected significantly less *Ae. albopictus* and *Ae. albopictus* females per trapping period (*P* < 0.001, *P* < 0.001), whereas no statistical differences were found between the HLC and BG trap (*P* = 0.970, *P* > 0.05). Statistically significant positive spatial correlations for *Ae. albopictus* sampling was found between HLC and HDN traps (*r* = 0.543, *P* < 0.001) and HLC and BG traps (*r* = 0.658, *P* < 0.001). In conclusion, both the BG trap and HDN have a significant positive spatial correlation with HLC, making them safer alternatives to HLC for *Ae. albopictus* monitoring in China. However, with better a sampling efficiency, being less labor intensive, and no human-baited attraction bias, the BG trap could be a better choice than the HDN trap.

## Background

Dengue fever is a high-risk vector-related infectious disease with rapid global transmission [1]. It is believed that the number of dengue cases worldwide has increased 30 times in the past 50 years, with 40% of the global population at risk. It is mainly distributed in tropical and subtropical Africa, the Americas, Southeast Asia, the Western Pacific and Europe [2–4]. Since 2010, most outbreaks have been concentrated in the western Pacific regions, such as Singapore, China, and Malaysia [5]. With the distribution range of *Aedes albopictus* in China continuing to expand, ≥ 168 million people are at high risk of dengue fever annually, which makes dengue fever a major public health threat in China [6, 7]. Without vaccines and effective medicine, monitoring and controlling arthropod vectors are important means of controlling dengue fever [7]. Mosquito surveillance has been regarded as the cornerstone for the development of mosquito control operations because surveillance information can guide control efforts and evaluate the efficacy of vector management [8]. *Ae. albopictus* is the only vector for transmitting dengue in Zhejiang province, as well as the main vectors responsible for dengue transmission in mainland China, [9, 10]. Monitoring *Ae. albopictus* is important for predicting the dengue outbreak risk and evaluating the effects of dengue epidemic control in Zhejiang, China. However, for *Ae. albopictus*, none of the existing traps without human attractants is as effective as the traditional human landing catch method (HLC) [11, 12].

HLC uses humans as attractants, and mosquitoes are collected when they land on exposed legs. At present, HLC is still considered as the “gold standard” because of its high efficacy in monitoring *Aedes* mosquitoes [9, 13]. However, when *Aedes*-borne diseases break, they pose a risk to field work professionals, because human attractants should continue exposing themselves to infective bites of known and unknown pathogens-bearing vectors. Thus, many new methods have been developed, such as the human-baited double net trap (HDN) and the BG trap [9]. The HDN consists of two box nets and uses human bait as a mosquito attractant. A large net contains a smaller net, and the outer net is raised off the ground to attract and collect mosquitoes between the two nets. People sit or stand in the inner net to attract mosquitoes, while others collect the mosquitoes between the two nets [9, 14]. HDN is much safer than HLC because the human-baiter is protected from mosquito landing and biting, and the outer collector can be protected by long-sleeved clothing and repellents [9]. Thus, HDN was recommended by China center for diseases control and prevention (CDC) for emergency monitoring when *Aedes*-borne diseases break out. However, HDN has been reported with low efficiency for capturing *Aedes* mosquitoes and few studies has evaluated its effectiveness of replacing HLC in China [12, 15]. The BG trap uses carbon dioxide (CO_2_) and an attractant that mimics human scent to attract mosquitoes, and once mosquitoes are attracted and fly to the trap, they are automatically caught [16]. In America, BG-Sentinel traps have been considered the “gold standard” for collecting *Aedes stegomyia* mosquitoes [17]. Although HLC has traditionally been considered the most effective method for monitoring highly anthropophilic mosquitoes, few evaluation studies have carried out to compared HLC with HDN and some other newly developed methods for *Ae. albopictus* collection in China.

In this study, we attempted to verify the effectiveness of HDN and BG traps on *Ae. albopictus* surveillance and their efficiency in sampling adult *Ae. albopictus* compared to that of HLC. We attempted to determine the relationships between these three methods for monitoring *Ae. albopictus* and to evaluate the effectiveness of HDN replacing HLC, at the same time to explore the potential of BG traps to replace HLC for *Aedes* monitoring.

## Methods

### Study sites

**Fig. 1:**
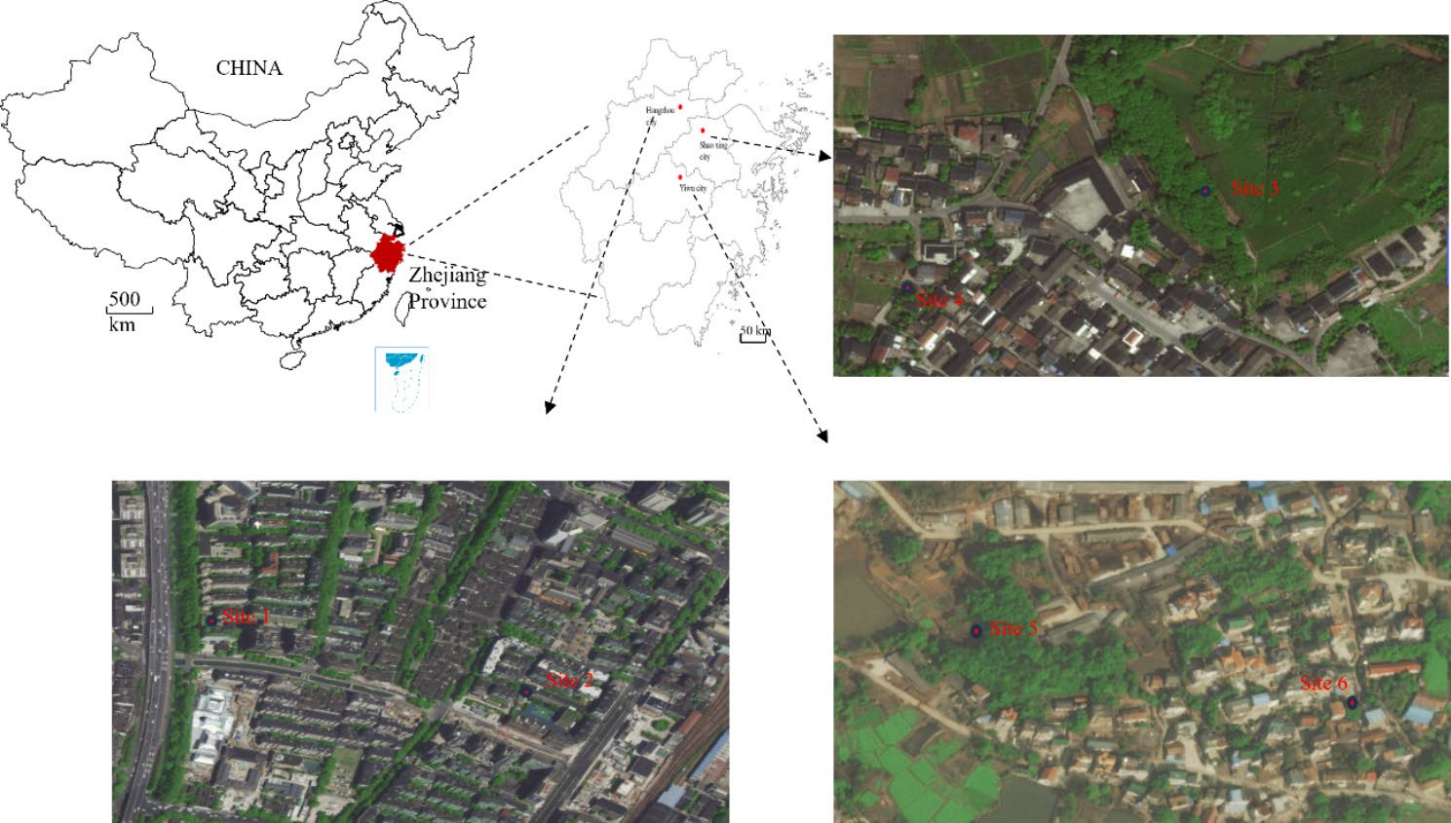
Locations of the six sites for mosquito monitoring comparison among three catches in Zhejiang province, China

This study was conducted on sunny and cloudy days from June to September 2021 in three cities where had ever experienced outbreaks of dengue fever in history. Hangzhou, Shaoxing, and Yiwu located in the north and center of Zhejiang Province, China, all of which have a subtropical monsoon climate, with a temperature ranged 20 ~ 34 ℃, rainfall ranged 91 ~ 228 mm from June to September 2021. Six field-monitoring sites (site one to site six) representing urban, suburban, and downtown environments were selected for mosquito sampling. The study was conducted in areas without reported local mosquito-borne diseases such as dengue fever, chikungunya fever, and Zika cases, before or during the study period in 2021.The details of the locations are shown in Table 1; Fig. 1.

**Table 1:** Geographical Information for the six Mosquito Sampling Sites

Site	City	Areas	Type of environment	Coordinates
Site1	Hangzhou	Urban	Residential neighborhood	30°15’43.74"N, 120°11’29.84"E
Site2	Hangzhou	Urban	Residential neighborhood	30°15’41.02” N,120°11’24.31” E
Site3	Shaoxing	Downtown	Green area	30°3’9.11” N, 120°22’24.59” E
Site4	Shaoxing	Downtown	Residential neighborhood	30°4’16.81” N, 120°22’3.86” E
Site5	Yiwu	Suburban	Park	29°17’58.34” N, 120°5’1.81” E
Site6	Yiwu	Suburban	Residential neighborhood	29°19’51.94” N, 120°2’54.43” E

### Study participants

Informed consent was obtained and 24 volunteers aged 23–54 years (13males and 11females) were recruited for participation. Training was carried out for all the participants before the study.

### Study design

**Fig. 2:**
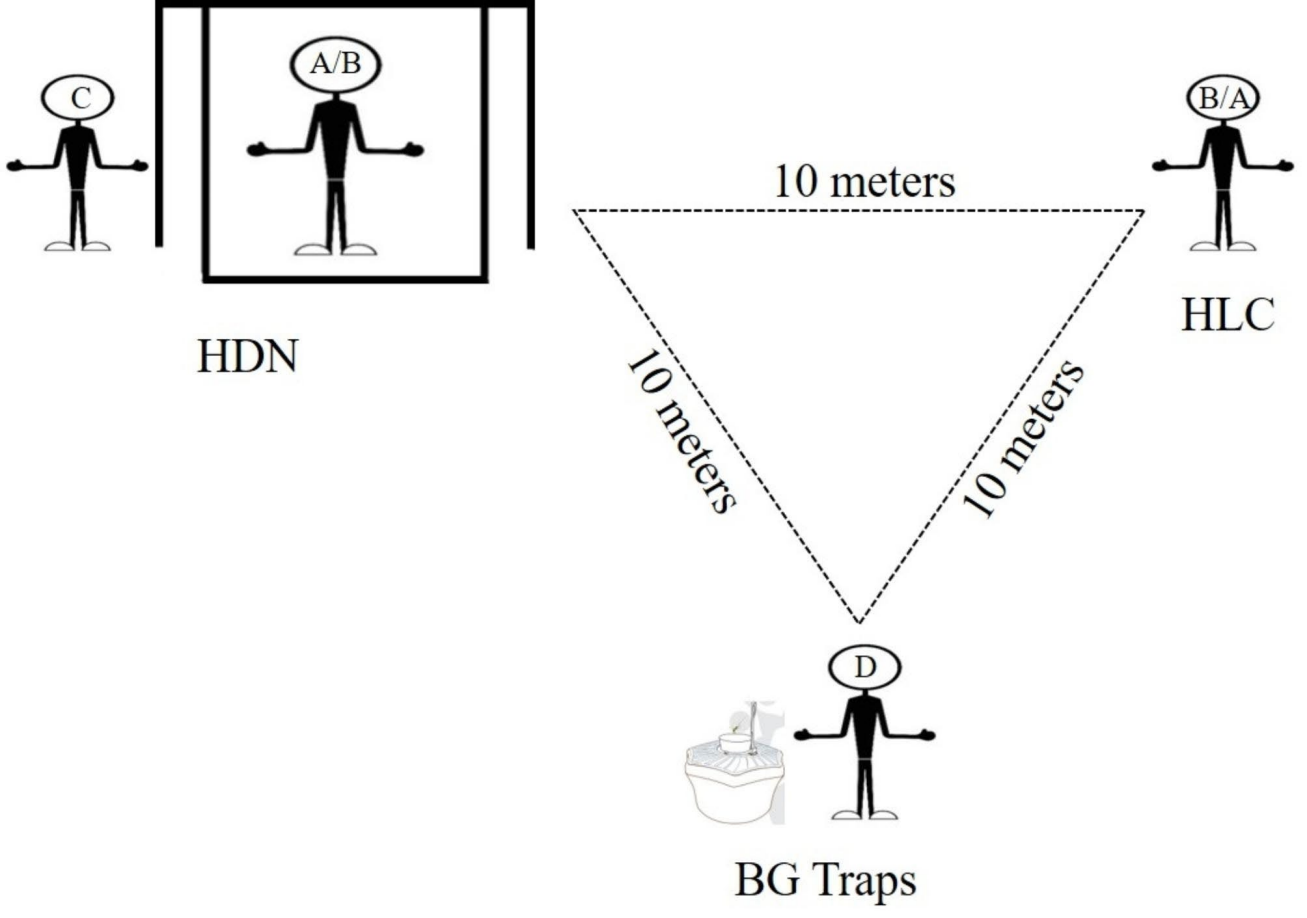
Field mosquito monitoring with three catches

**Fig. 3:**
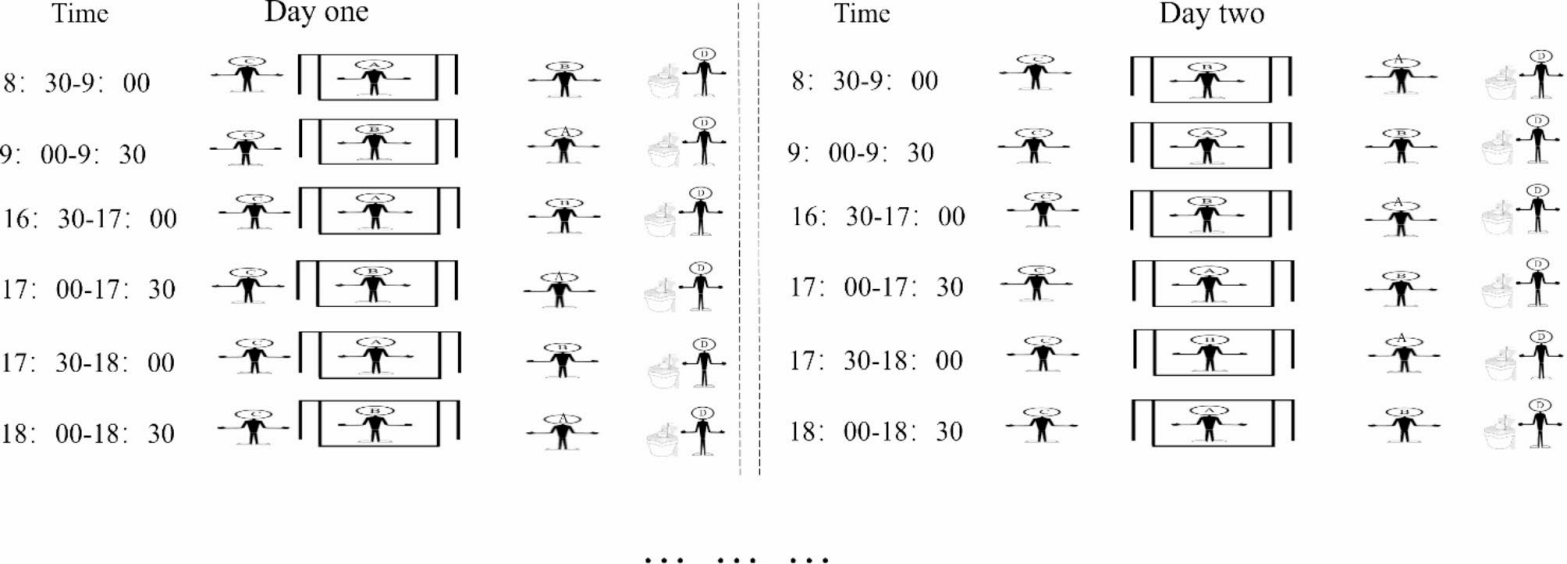
Comparison among three catches at each site

The catches were performed three times a day in peak period of *Ae. albopictus* activity in Zhejiang province, China. Each session lasted for one hour. These were 8:30–9:30 AM, 16:30–17:30 PM, and 17:30–18:30 PM, representing morning, afternoon and evening. At each site, three catches were performed simultaneously, 10 m apart (Fig. 2) [18]. The 24 volunteers were randomly separated into six groups, and each group containing four participants (from A to D) was responsible for three catches at one site. Participants A and B were designated human baits to lure mosquitoes in the HLC or HDN catches. Participant C was responsible for collecting mosquitoes in the HDN catch, cooperating with participants A or B. Participant D was responsible for capturing mosquitoes with the BG trap. To minimize the attractant bias, human baits A and B of HLC and HDN were exchanged between the first and second 30 min of each time, while participants C and D always acted as collectors in HDN catches and operators in BG-trap catches, respectively (Fig. 3). In each site, the capture was carried out in two sunny or cloudy days with similar climate factors that are at least 14 days apart according to the weather forecast. To minimize the possible time bias between the first and second 30 min, participants A and B changed orders in HLC and HDN catches the next day. The per-trapping period lasted for 30 min. The details are shown in Fig. 2.

### Mosquito sampling

#### Human landing caches

The participant who performed the human landing catch exposed his right leg and collected mosquitoes landing on his leg (his left leg was protected by long pants), using a portable battery-powered aspirator.

#### Human-baited double net trap (HDN)

The patented double net trap developed by the Chinese Center for Disease Control and Prevention was used, with an outer net measuring 180 cm long, 180 cm wide, and 150 cm high and an inner net measuring 120 cm long, 120 cm wide, and 200 cm high. The bottom of the outer net was raised 35 cm above the ground, whereas the bottom of the inner net was hanging to the ground. One human baiter sat inside the inner set with two legs exposed, and one collector with long-sleeved clothing collected attracted mosquitoes between the two nets. No repellent was used by the human baiter or collector during the study period.

#### BG trap

BG traps (version: BG-Mosquitaire CO2) developed by the BioGents GmbH Company (Regensburg, Germany, SN:00040145) were used in this study. A black funnel trap was placed on the ground, with the trap mouth opening upward. BioGents GmbH Company’s self-developed mosquito attractant was put in the funnel trap, the power supply was connected, and the carbon dioxide valve was opened, with a carbon dioxide flow of 0.3 L/min.

Collected mosquitoes were taken to the Zhejiang Provincial Center for Disease Control and Prevention Laboratory, killed by freezing, and identified using taxonomic keys [13].

### Statistical analysis

Statistical analyses were performed using Statistical Package for the SPSS (version 23.0) [19]. Generalized linear mixed models (GLMMs) were used to analyze the effect of different catches on the total number of *Ae. albopictus* (both male and female), and *Ae. albopictus* females caught per monitoring period (30 min), based on negative binomial regression. The dependent variables were modeled via GLMMs controlling for independent random variables (“days,” in this case) to test the statistical significance of fixed independent variables (“catches,” “sites,” “time” and “baits”). The means and standard errors associated with GLMMs were calculated. Pearson correlation analysis was used for spatial sampling yields between HDNs and HLCs and between BG traps and HLCs. Statistical significance was set at *P* < 0.05.

## Results

A total of 2330 adult mosquitoes were captured, including 2274 *Ae. albopictus*, 52 *Culex pipiens* complexes *(*mainly *C. quinquefasciatus and pallens)* and 4 *Armigeres subalbatus*. *Ae. albopictus* and *C. pipiens* complex were collected from all three catches, whereas *A. subalbatus* adults were caught by HLC and BG traps only. The species and sex composition of the adult mosquitoes captured among the three catches are shown in Table 2. *Ae. albopictus* was the most abundant species collected by all three catches, with 848 (98.95%), 559 (97.39%) and 867 (96.44%) caught in HLC, HDN and BG traps respectively. Only 8 (0.93%), 15 (2.61%), and 29 (3.23%) *C. pipiens* complexes and 1(0.12%),0 (0.00%), and 3(0.33%) *A. subalbatus* were collected using HLC, HDN, and BG traps, respectively. More female than male mosquitoes were captured by HLC (80.86% vs. 19.14%), HDN (66.55% vs. 33.45%), and BG traps (70.52% vs. 29.48%) (Table 2).

**Table 2:** Mosquito species and sex composition captured using HLC, HDN and BG traps

Collection methods	*Aedes albopictus*		*Culex pipiens* complex		*Armigeres subalbatus*	
	Female n (%)	Male n (%)	Female n (%)	Male n (%)	Female n (%)	Male n (%)
HLC	687(81.01)	161(18.99)	5(62.50)	3(37.50)	1(100.00)	0(0.00)
HDN	368(65.83)	191(34.17)	14(93.33)	1(6.67)	0(-)	0(-)
BG trap	606(69.90)	261(30.10)	25(86.21)	4(13.79)	3(100.00)	0(0.00)
Total	1661(73.04)	613(26.96)	44(84.62)	8(15.38)	4(100.00)	0(0.00)

**Fig. 4:**
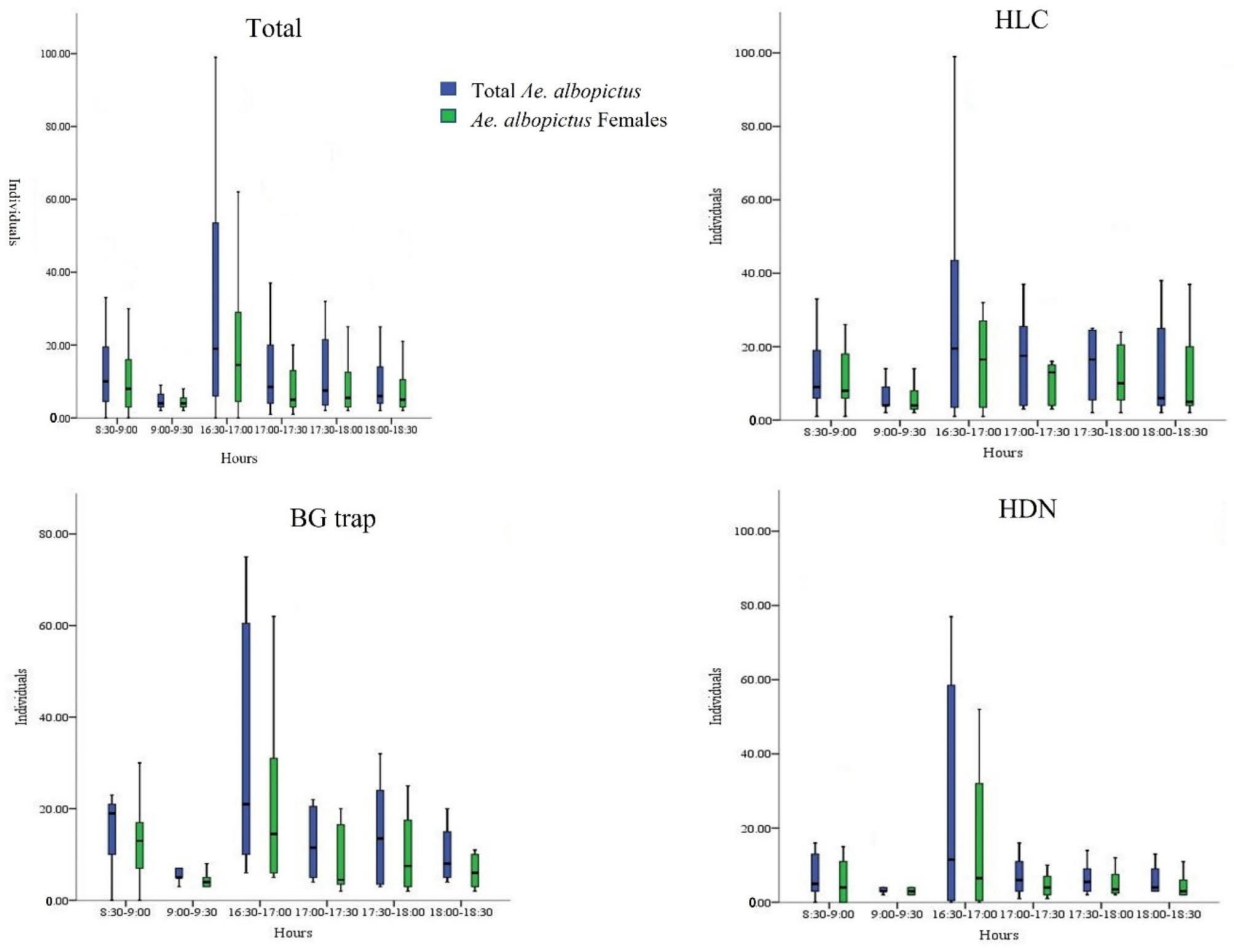
Difference in the number of mosquitoes captured at different times of the day

Statistically significant variations in total *Ae. albopictus* catches were found during the different hours of the day (GLMM, F _(5,122)_ = 8.440, *P* < 0.05), and more *Ae. albopictus* tended to be caught at 16:30–17:00 PM (Fig. 4). For different catches, the same hourly variations in *Ae. albopictus* were presented in the HLC (GLMM, F _(5,23)_ = 4.878, *P* < 0.05), HDN (GLMM, F _(5,23)_ = 6.240, *P* < 0.05), and BG traps (GLMM, F _(5,40)_ = 4.323, *P* < 0.05) (Fig. 3). In the present study, 86.53% (45/52) *C. pipiens* complex and two *A. subalbatus* were trapped after 17:00 PM. No human bias was found in this study (GLMM: F _(18,122)_ = 0.945, *P* > 0.05).

Significant differences between total number of *Ae. albopictus* adults and *Ae. albopictus* females alone were detected in the three catches (GLMM, *Ae. albopictus*, F _(1,122)_ = 14.293, *P* < 0.05; *Ae. albopictus* females, F _(1,122)_ = 28.759, *P* < 0.05). Compared to HLC, HDN collected significantly less *Ae. albopictus* and *Ae. albopictus* females per trapping period, whereas no statistical differences were observed between the HLC and BG traps (Table 3). The sampling efficiency of the HDN and BG traps for *Ae. albopictus* were approximately 0.66 and 1.02 times that of HLC, respectively, and for *Ae. albopictus* females, the sampling efficiencies were 0.54 and 0.88 times that of HLC, respectively (Table 2). Though the mean *Ae. albopictus* catch by HDN was significantly lower than that by HLC, a significantly positive spatial correlation between HLC and HDN for *Ae. albopictus* and *Ae. albopictus* female was found (*Ae. albopictus*: *r* = 0.543, *P* < 0.001; *Ae. albopictus* females; *r* = 0.694, *P* < 0.001). A positive spatial correlation between the HLC and HDN was also detected (*Ae. albopictus*: *r*_(51)_ = 0.658, *P* < 0.001; *Ae. albopictus* females; *r* = 0.669, *P* < 0.001).

**Table 3:** Differences of the number of individuals captured per trapping period among three catches

Catches	*Ae. albopictus*				*Ae. albopictus* Females			
	Estimate	SE	t	*P*	Estimate	SE	t	*P*
HDN	-0.490	0.130	-3.781	< 0.001^*^	-0.696	0.130	-5.363	< 0.001^*^
BG trap	-0.019	0.488	-0.038	0.970	-0.424	0.474	-0.895	0.373
HLC^#^	0	/	/	/	/	/	/	/

Mean +/− SE differences in the least squares means associated with the mixed linear models for the number of individuals per trapping period among three catches. Estimate: differences in the least squares means, SE: standard error, t: t-value, p: p value

#HLC was selected as the baseline

*Significant differences were found

## Discussion

HLC, HDN, and BG traps are widely used to monitor adult mosquitoes worldwide. The results of the present study indicate that the individuals of *Ae. albopictus* caught by both the HDN and BG traps were positively correlated with that caught by HLC. The *Ae. albopictus* sampling efficiency of HLC was significantly higher than that of HDN, which was statistically similar to that of the BG trap. This result is highly consistent with the comparisons conducted between HLC and HDN by Gao et al. in Shanghai [14].

Zhejiang is located in southeast China and *Ae. albopictus* was the only vector responsible for *Aedes*-borne diseases such as dengue fever and chikungunya fever. Thus, the subjects in this study were *Ae. albopictus*, particularly *Ae. albopictus* females because only female mosquitoes would take blood and transmit diseases, the role of which is crucial in monitoring [20]. Based on the results of this study, the number of *Ae. albopictus* and *Ae. albopictus* females collected by HDN were significantly lower than those collected by HLC (both *P* < 0.001). Compared to HLC, only 0.66 times of *Ae. albopictus* and 0.54 times of *Ae. albopictus* females were collected by HDN, which was similar to the results from Shanghai, Uganda, and Nigeria studies, with individuals of *Ae. albopictus* captured by HDN being 0.4, 0.5 and 0.25 times that of HLC, respectively [14, 21, 22]. This might be attributed to the trap design of the two box nets in the HDN, which limited excessive attractive emanations from the hosts inside the inner net. This suggests that HDN may underestimate *Ae. albopictus* during the monitoring period. Too moderate a sampling efficiency would also cause HDN to fail in distinguishing the effect differences of *Aedes* mosquito management and fail in guiding control efforts when *Aedes*-borne diseases break [15].

Owing to the relatively small sample size, no statistically significant variation in human baits was found in this study. However, human-baited bias existed in traps that used humans as attractors, as previously reported [14, 23]. The reason might be that heat, water vapor, CO_2_, and various odors that lure mosquitoes emanating from different individuals differed [23]. HDN was supposed to reduce human-baited bias because of the design of the two box nets limiting attractive emanations, which is also the reason for its low sampling efficiency. The dilemma of HDN is that the design of double nets has lost its efficiency in attracting mosquitoes and requires more labor to make it safer and less bias to human bait. The BG trap has been used for *Aedes*-mosquitoes monitoring in North America, Singapore, and Australia [24–26]. As there were no human baits used, the results would be more comparable among different locations.

According to previous research, the CO_2_ flow in the BG traps was set to 0.3 L/min in this study, which was considered to be the most appropriate for *Ae. albopictus* monitoring [19]. Sampling efficiency of BG traps for *Ae. albopictus* and *Ae. albopictus* females were statistically similar to those of the HLC. Similar results were reported by Krockel et al. in Brazil [27]. In addition, compared to HLC and HDN catches, the BG trap method could save more labor. One field professional could operate several BG traps to monitor the mosquito density in several places at the same time, while using HLC or HDN catches would require one or two laborers to be in one place for at least 30 min.

This study was conducted during the peak biting periods of *Ae. albopictus*, and more mosquitoes (mainly *Ae. albopictus*) tended to be caught at the first half hour (16:30 − 17:00), it might be caused by the bloodbucking habits of *Ae. albopictus* on one hand. On the other hand, it might be caused by continuous capture in the afternoon/evening, because mosquito density might be reduced due to repeated capture. Secondly, the distance between catches were set within 10 m by some previous researches when comparing their efficiency in capturing mosquitoes [9, 12, 28, 29]. An pre-experiment was also conducted in this study, and the results showed that the density of *Ae. albopictus* was more probably remaining consistent within a range of 10 m (unpublished data). Thus 10 m were set apart between each catch to balance between reducing physical interference among three catches and the consistency of mosquito density in the environment where three catches were located. But the interference among catches might not be removed thoroughly. Therefore, caution should be exercised when extrapolating all these results. Besides, the research object was only *Ae. albopictus*. In the future, studies should be conducted to explore the relationship between different monitoring methods on other mosquitoes, such as *Anopheles* and *Culex* species in China.

## Conclusions

With a significantly positive spatial correlation with HLC, both the BG trap and HDN could be safer alternatives to HLC for *Aedes albopitus* monitoring in China. Because BG traps have better sampling efficiency, are less labor-intensive, and do not have a human bait attraction bias, they might be a better choice than HDN traps.

## Data Availability

All data generated or analysed during this study are included in this published article.
